# A potential novel option for cancer immunotherapy – TLR7 stimulation inhibits malignant melanoma bone invasion

**DOI:** 10.18632/oncotarget.25872

**Published:** 2018-08-07

**Authors:** Takenobu Katagiri

**Affiliations:** Takenobu Katagiri: Division of Pathophysiology, Research Center for Genomic Medicine, Saitama Medical University, Saitama, Japan

**Keywords:** malignant melanoma, bone invasion, toll-like receptor, cancer immunotherapy

Malignant melanomas show quite high levels of malignancy and poor prognosis in many affected patients due to metastasis, but they occur less frequently than other cancers. Various methods have been attempted for treating malignant melanomas, including surgery, chemotherapy and radiotherapy; immunotherapy has been used effectively in recent years, and it has fewer side effects and a sustained therapeutic effect compared to other treatment options.

Immunotherapy for malignant melanoma was originally established several years ago following the isolation of melanoma-associated antigen (MAGE) 1 and the finding that MAGE1 was recognized by tumor-specific cytotoxic T lymphocytes (CTLs) isolated from a patient with malignant melanoma in 1991 [1]. Since then, several different cancer antigens and their epitopes were shown to be recognized by CD8^+^ T and CD4^+^ T cells [2]. More recently, some biological inhibitors of immune check points, such as anti-CTLA-4 and anti-PD1/PD-L1 antibodies, were shown to be effective for treating various types of cancers, including malignant melanoma [3, 4]. However, a major drawback of these new biological therapeutic agents is the economic burden on the patients and/or families.

Toll-like receptors (TLRs) recognize molecule-derived pathogens and function as a biological defense by activating the immune system. Therefore, TLRs are potential targets for anti-cancer therapy because they utilize the immune system activation capacity [5]. In this issue of *Oncotarget*, Manome et al. report an interesting malignant melanoma treatment option, R848, which is a small chemical agonist of TLR7. The authors found that treating mice with R848 suppressed malignant melanoma bone invasion without directly suppressing proliferation; this finding suggests that an indirect mechanism is involved in this effect of R848. Moreover, the authors found that treating bone marrow macrophages with R848 in vitro induced the expression of pro-inflammatory cytokines, such as IL-6, IL-12 and IFN-γ. Conditioned media stimulated with R848 inhibited malignant melanoma cell proliferation, and this inhibition was ablated by adding a neutralizing antibody against IL6, IL-12 or IFN-γ. This finding suggests that TLR stimulation with agonists, including TLR7 by R848, is a potential novel option for cancer immunotherapy for advanced metastatic malignant melanoma.
